# Ten simple rules for effective research data management

**DOI:** 10.1371/journal.pcbi.1013779

**Published:** 2025-12-08

**Authors:** Max J. Hassenstein, Klaus Jung

**Affiliations:** 1 Institute of Animal Genomics, University of Veterinary Medicine Hannover, Foundation, Lower Saxony, Germany; 2 Unit for Research Data Management, University of Veterinary Medicine Hannover, Foundation, Lower Saxony, Germany; Carnegie Mellon University, UNITED STATES OF AMERICA

## Introduction

Advances in information technology, digitalization, database volume, the internet, high-throughput measurement technology, and artificial intelligence (AI) have profoundly transformed research. In the 20th century, it was common for a study or an experiment to yield one single file (e.g., a table). Today, many research projects yield many files, often created by multiple collaborators and are often valuable for secondary use. For instance, experiments in genomics [1] the field of work of one of the authors, can generate multiple raw files per biological sample, plus processed data. Furthermore, scientific knowledge is currently generated not only through hypothesis-driven statistical inference but also using (un)supervised data mining and AI techniques [2] applied to existing resources. These developments require effective research data management (RDM) at both project and institutional level, i.e., institutional RDM policies and infrastructure provision, to facilitate collaboration, data and model reuse—e.g., for transfer learning—and compliance to fundamental guidelines such as the good scientific practice [3–5].

**Fig 1:**
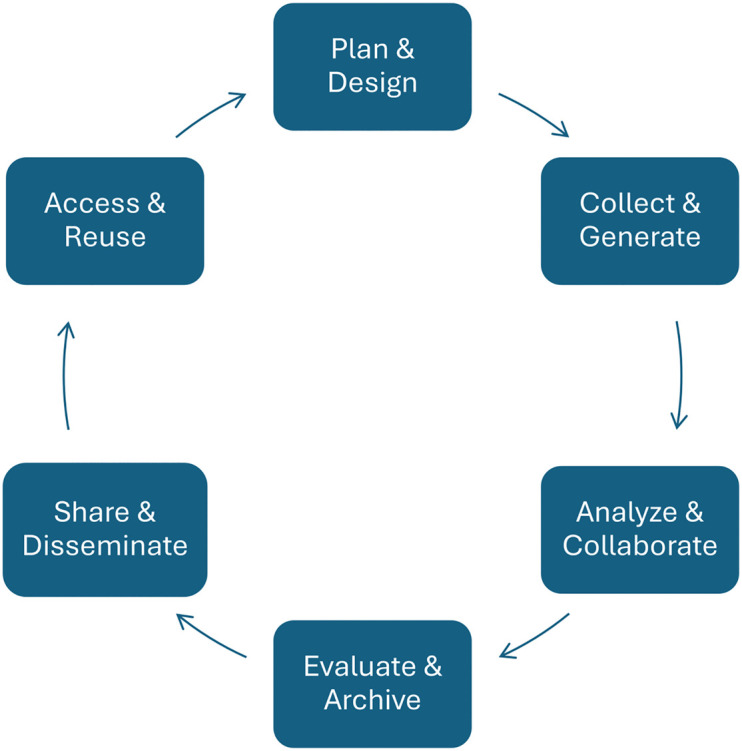
Data life cycle. Suggested by Surkis and Read [7], own illustration.

Research data are typically those collected by a research group to address a scientific problem and contributed to solve it [6]. RDM broadly encompasses all data-related activities throughout the data life cycle (Fig 1).

Moreover, RDM is critical when the conventional sequence of research—from hypothesis formulation, data collection, and analysis, to hypothesis testing—is replaced by analyzing accumulating data [8,9]. Furthermore, RDM is crucial in research synthesis such as meta-analyses based on published results (i.e., secondary analysis) have become common, but so have those based on available raw data [10–12].

Poorly managed data can lead to various problems, such as analysis errors or lost reuse opportunities. A prominent historical example is Christopher Columbus encountering the Americas while intending to reach Asia—simply put because his calculations relied on data that significantly underrepresented the Earth’s true size [13].

Several publications of the “Ten Simple Rules” series offer guidance on RDM subdomains, such as *Ten Simple Rules for Creating a Good Data Management Plan* [14], *Ten Simple Rules for Reproducible Computational Research* [15], or *Ten simple rules for large-scale data processing* [16]. However, they focus on project-related RDM topics. Certain institutional-level or holistic RDM matters remain unaddressed. Universities and research institutes increasingly seek to store data from individual projects systematically for reuse. Thus, our *Ten Simple Rules for Effective Research Data Management* provide a condensed reference of significant RDM topics applicable at higher organizational levels, arranged in a logical sequence corresponding to the research data life cycle. The rules are derived from the authors’ diverse expertise in statistics, genomics, bioinformatics, public health, epidemiology, and research data management consulting. They may serve as a reference for institutions, researchers or professionals regardless of field or career level. In the supplementary material, researchers can find a table with useful RDM tools (S1 Table).

While the ten rules outline established principles in research data management, their implementation often encounters challenges. This work also addresses such key problems exemplarily, offering practical strategies to enhance applicability.

## Rule 1: Consider applicable policies

Policies, guidelines, and compliance measures shape the way how researchers work, including how data are managed—from data collection, storage to sharing. These therefore affect both RDM activities and research outputs.

Countries, umbrella organizations, science foundations, professional societies, institutions, working-group leaderships, funding bodies, and project boards may issue such policies and guidelines [5,17–21]. These inherently reflect best practices, outline legal issues, or offer suggestions for efficiency and resource management. Such guidelines may be published by their issuers to articulate a position on certain topics and promote behaviors in the target group. Typical topics include research integrity, e.g., promoting transparency and reproducibility, data handling or safeguarding, or access to research data and results, especially for publicly funded work.

Researchers should check for applicable policies and compliance requirements in their subject area, as well as any peculiarities specific to their individual research. If certain policies or compliance rules most likely apply to the work, researchers should try to understand their focal areas and general goals, e.g., transparency mandates, and follow them as reasonably as possible. To gather policy requirements, consult funder websites, institutional research support offices, or data management plan (DMP) tools (see rule 7). For example, the DMP tool RDMO (S1 Table) includes forms tailored to various funder requirements, thereby facilitating adherence.

Awareness of, and adherence to these requirements is essential for effective RDM, as misguided processes, corrections, and or later amendments require additional and unnecessary resources [22]. A common challenge is anticipating potential data usage early, before or during collection, to align with policies. In the context of data governance, the researcher can map potential data publication scenarios at project initiation (e.g., public versus closed or controlled access) against funder or legal rules, such as the U.S. National Science Foundation (NSF) or German Research Foundation (DFG) guidelines. If no such guidelines exist or apply to the work directly, consider global guidelines such as the *International Ethical Guidelines for Health-related Research Involving Humans* [23] or the Statements of the *World Conferences on Research Integrity* [24]*.* Composing a data policy may be beneficial for larger research projects or those with data peculiarities requiring certain handling [25–27].

## Rule 2: Account for ethical regulations and legislation

Ethical regulations and legislation shape how researchers conduct research. They affect critical RDM activities such as data collection and publication [28]. At any given time, both ethics and law must be adhered to with respect to the rights of individuals, protect their data and privacy, and preserve property rights. Although policies and guidelines (see rule 1) may inherently refer to ethical principles and applicable laws, it is the responsibility of the researcher to adhere to all applicable ethical principles and laws, including those potentially unheeded by those guidelines. Thus, considering ethics and law will help researchers avoid issues over the course of their work. Being aware of legal boundaries and acting ethically will ensure that projects can proceed as planned without pitfalls, such as those requiring protocol changes, or even dropping certain aspects—including those to which significant resources have already been dedicated to. Therefore, this rule may help avoid later nuisances such as manuscript rejections from publishers due to ethical considerations, or more considerable consequences such as punishable offenses.

Relevant legislation typically pertains to data collection and sharing, designed to safeguard personal data of individuals and to facilitate informational self-determination. For example, within the European Union (EU), individuals possess the right to know which information is collected, processed, and transmitted (General Data Protection Regulation, GDPR).

Ethics committees ensure that research is conducted ethically and in accordance with responsible research practices. These committees review research proposals for ethical compliance, aiming to ensure the rights and safety and well-being of participants. Determine early whether a study requires an audit by an ethics committee; if so, incorporate sufficient response times into the project planning. Be aware that protocol amendments may become necessary, and these may require re-review by the committee.

We acknowledge that requirements may change rapidly, such as the EU Data Act or updates to the GDPR, which can render active DMPs invalid. Regular review and consulting with professionals, e.g., the institutional legal department, can help researchers to be mindful of necessary adjustments.

## Rule 3: Have a sound data storage and security concept

Storing files on a modern computer is easy. However, securing them over time and in a sustainable way, i.e., avoiding data corruption data loss, requires following some simple principles. While a DMP (rule 7) may declare how to administer and store project-specific data, researchers will manage “everyday data” (e.g., non-project-specific files) that span projects—such as manuscripts, project funding applications, or code libraries.

First, avoid storing any valuable data exclusively on a single computer or storage device, as it represents a single point of failure. Ideally, valuable data should be stored in accordance with the *3-2-1 backup rule*: keep three separate instances of the data—the original and two backups—on two distinct storage devices, such as a local copy on a laptop plus network storage, and one offsite backup at a location different from the current one.

For those working at an institution, access is likely available to its closed institutional network, including network drive access. Such network drives are typically backed up automatically. Check with the institution to confirm if and how the data are backed up locally and how to restore it in case of a local storage failure. If no backup strategy exists, act accordingly and add sufficient layers of data protection, such as external hard drive backups. This is especially relevant for researchers in the field or traveling with restricted network access.

However, a sound data storage concept is not just about avoiding data loss. Data might be valuable and sensitive, meaning not everyone ought to it. Therefore, regulate access rights for authorized personnel and ensure that access rules are enforced within the IT infrastructure. Also, consider encrypting sensitive data, especially on external drives—tools such as VeryCrypt (see S1 Table) can facilitate this.

It is best practice to retain research data over time. Data retention requirements may vary by country, funder, data type, or subject domain, but may require a minimum of 10 years, ranging up to over 25 years [5,29]. Estimated storage requirements should be coordinated with the IT department and may be included in application documents (see rule 7, DMP).

In general, IT resources for RDM should follow a long-term concept. With the increasing amount of data accumulating in individual research projects, some universities struggle to provide sufficient storage capacities. When applying for project funding, researchers should therefore also clarify with their IT department whether sufficient resources are available or whether additional funding should be applied for to increase capacities.

We recognize that small or independent researchers may lack access to advanced IT infrastructure for automated, secure, and scalable data backups. In such cases, manual processes for backing up and securing data, such as managing multiple hard drives for timely backups or handling passwords for encrypted data, are prone to human error. To support compliance with this rule, researchers should outline these processes in their DMP (see also rule 7, DMP).

## Rule 4: Keep your working directories clean

**Fig 2:**
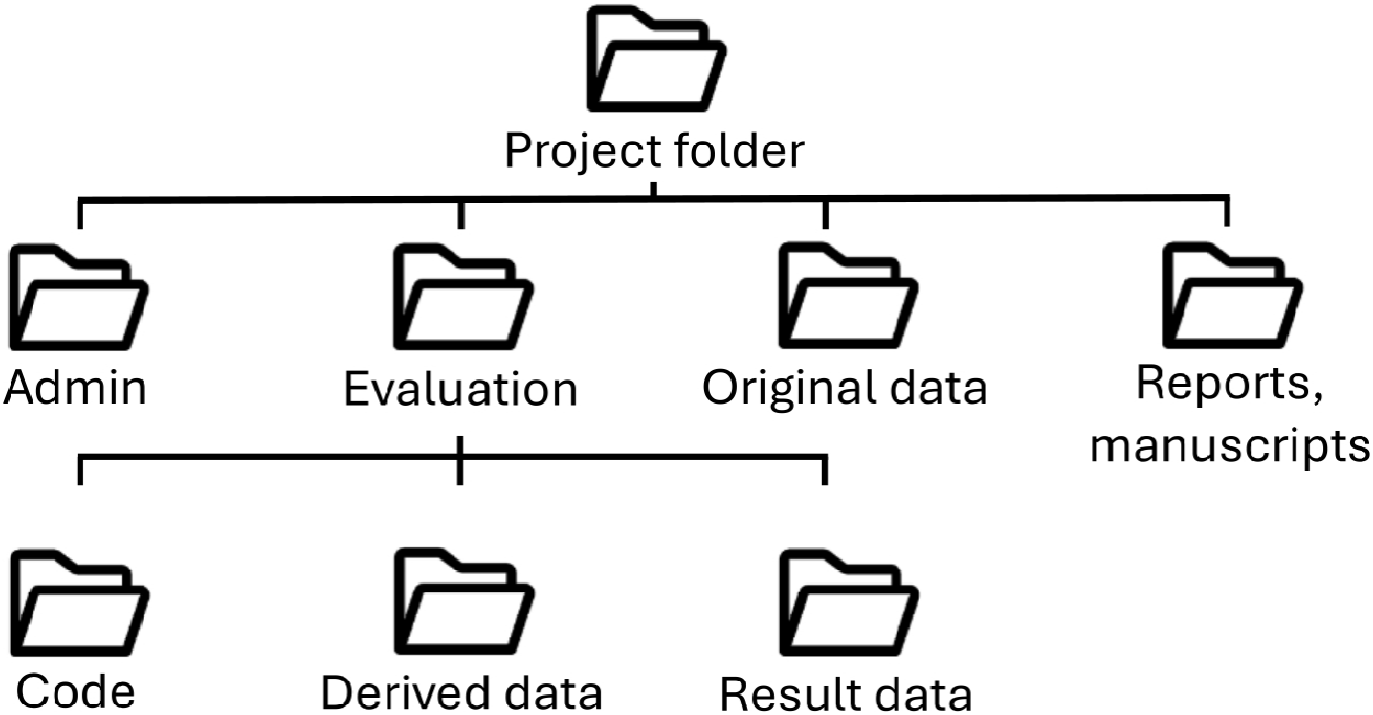
Exemplary folder structure. The project folder ideally contains all project files in its logically subdivided folders. Using a similar folder structure across projects can facilitate data retrieval and promote standardization, while allowing for variations as necessary per project.

A systematic organization of files and working directories is key to efficient filing, navigation, and prompt file retrieval. Using the same filing scheme for files and directories across projects and teams can simplify and accelerate interactions. Clean working directories have two characteristics: first, they have a logical and uniform structure for file directories, i.e., a standardized folder structure and depth, as well as default folder names (Fig 2).

**Fig 3:**
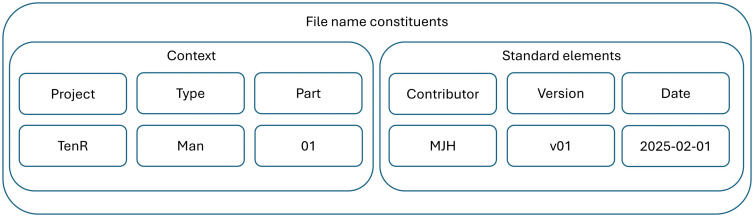
Exemplary file naming structure. Each file name can be broken down into its constituents, context, and standard elements. The context provides information on the project, document/data type and, if applicable, part. The standard elements consist of the main contributor, version, and date. This logic may be altered by preference or specific requirements.

Second, files adhere to a consistent file naming convention, which facilitates understanding of what is within the file at a glance (Fig 3).

Both folder and file naming structures above are exemplary. By following the schema, a file name might be “TenR_Man_01_MJH_v01_2025-01-01.docx. Researchers can adjust it to both their institutional, project-related, or personal requirements. What is crucial is to establish these as a standard and consider them in every new project. We recognize that it is not always feasible to apply a new scheme to old directories or files, but the researcher should try to implement it in all prospective projects.

As we suggest a global folder structure and file naming scheme, apply it wherever possible, whether on a working computer, institutional network drives, or shared cloud folders.

We recognize that this basic rule, though frequently overlooked in practice, can impede file retrieval or collaboration. Initiating each new project with a standardized folder structure promotes organization from the start. Additionally, various tools are available for batch file renaming, which streamline rule adherence (S1 Table).

## Rule 5: Have a sound data collection strategy

To reach well-founded conclusions, researchers require quality data. Outlining a data collection methodology is one of the first steps in conducting research, along with formulating the research question. Understand how the research question translates into specific data needs: what data are required, and what insights are expected from them, and in which way.

First, research published data to examine whether it can be integrated into the work. To find reusable data, skim abundant resources such as general or domain-specific data repositories at re3data (https://www.re3data.org). Reusing data can be a tremendous benefit and save significant resources, especially labor and material costs, as well as data retention costs in projects with high data volumes.

If new original data are required, the guiding principle is to collect as much as required, but no more than necessary. Depending on the research question, consider running a sample size calculation before collecting data to ensure collection of the minimum amount of required data [30]. Collected data beyond the requirements require additional resources, such as administration, data management, processing, and storage.

Next, choose an appropriate method aligning with the study’s objectives. Quantitative data collection is useful when requiring numeric data, which can be collected with questionnaires, observations, or experiments. When aiming to understand contexts and reasons, collect data with interviews, focus groups, or case studies. In some instances, both may be needed; subsequently, tailor the data collection to those needs. It is advisable to run a small pilot study to ensure that the intended data is collected or generated. Best practices for data quality may vary by discipline; for example, in social sciences, validation through triangulation is common, while in physics, calibration of instruments ensures accuracy.

Even the most sophisticated methods may not be capable of compensating for bias or lacking data quality introduced during data collection. Therefore, data quality assessment of both reused and original is vital. Fundamentally, data quality is the degree to which the data at hand can meet their intended purpose while being error-free [31]. The goal of data quality efforts is to assure that the data depicts the real-world entities they measure as comprehensively as possible.

Some research, such as meta-analyses, does not require original data collection but instead involves reusing existing data for further analysis. In these instances, it is equally essential to establish clear criteria for the required data and its relevant properties that must be met for reuse.

## Rule 6: Work on comprehensive documentation

Orderly and standardized documentation of both data and its collection method is key to understanding and using any kind of data, whether one’s own data or that of others. Data are usually not self-explanatory: with sufficient documentation, the work remains transparent and reproducible while being less likely to be misinterpreted.

Metadata–data about data—is an integral part documentation. Metadata comprises, for example, information on when data were created by whom and with which method. Furthermore, metadata may include file sizes, formats, and languages, such as the spoken language for text documents or programming language for code. Metadata may come in various forms–common forms are readme-files (Box 1), data dictionaries [32], or computer-readable XML/JSON files. A commonly recommended metadata schema is Dublin Core (www.dublincore.org).

**Box 1. Key aspects to include in a readme-file for metadata.**

**Table:** 

• Explanation of data included
• Original purpose of data included/project affiliation
• Author(s)/Creator(s)
• Date/period of data creation
• Required or recommended software/other particularities

Readme-files are the most basic form of documentation, and more complex data will require more extensive documentation. For instance, audiovisual data, a descriptive text may be useful, for tabular data, an orderly data dictionary can explain rows and columns, data types, and value ranges. Formats such as XML facilitate data description and are commonly used for metadata provision.

We observe that some metadata standards may not fully capture all relevant information in specific research domains. General frameworks, such as Dublin Core, facilitate the communication of standard details like author, date, or language but may lack labels to comprehensively describe data, such as study type in health sciences (e.g., cross-sectional, case-control, or cohort). In such cases, extending present metadata schemes becomes necessary. However, this introduces the challenge that proposed new metadata sets must first gain recognition and adoption to become established standards.

We emphasize the significance of incorporating funding information into the data documentation to promote transparency. Funding details provided solely in the original publication may prove inadequate, as they are often not sufficiently visible to data reusers who have no interest in the associated journal article.

## Rule 7: Develop a data management plan

A DMP essentially outlines data collection, storage, and sharing strategies while describing how privacy, security, and policy compliance are ensured. Usually, the DMP is drafted along with the project outline and accompanies the project throughout its lifecycle. Thus, a DMP can maximize the data’s value and impact. Drafting a DMP may seem like additional work, but its additional organization boosts efficiency and resource management. We recognize that a simple rule cannot do justice to a DMP as a whole, and *Ten Simple Rules for Creating a Good Data Management Plan* [14] provides a helpful overview of its most critical aspects.

There is no common standard for the topics and questions to be covered in a DMP, as they are shaped by potential specifications from professional societies, funders, and institutions. However, there are typical guiding questions to be covered in a DMP (Box 2).

**Box 2. Guiding questions for describing research data handling in a data management plan. Based on the German Research Foundation checklist for the appropriate handling of research data [33].**

**Table:** 

Collection: What data will be re-used, collected, or created, and how? Which processing steps are required?	Access: Who required access to the data during the project and does the access require access rules and authorization?
Description: What are the data formats/types to be collected, and to which extent? Which hardware/software is required?	Sharing and re-use: How will the data be shared and which conditions apply (e.g., licenses)? If the data cannot be shared or partially, why?
Standards: How will the data be described to allow an effective interpretation and use? Which standards apply (e.g., metadata standards)?	Roles & responsibilities: Who is responsible for the individual data-related steps (e.g., data collection, quality control, publication)?
Policies, legal & ethics: Which policies and funder requirements apply? How will legislation and ethical compliance be met?	Budget: Which financial implications arise from the data (e.g., hardware, storage, and/or software requirements)?
Storage & preservation: How will the data be stored, secured, and preserved, both during the project and long-term?	Quality control: How will data quality be ensured and monitored?

Funders play a pivotal role in RDM by mandating DMPs as part of grant applications, promoting data sharing, and enforcing retention policies (e.g., funders increasingly demand DMPs for grants). Free DMP tools (see S1 Table) can assist in creating compliant plans. While researchers can draft DMPs individually, involving institutional management ensures alignment with broader resources.

A common pitfall is that researchers may create a DMP initially—or, in some cases, neglect to draft one entirely—and fail to regularly review and update it to incorporate justified protocol changes or monitor adherence to formerly defined requirements. This oversight can lead to consequences ranging from increased workload to data mismanagement.

## Rule 8: Make use of standard and open file formats

Open and standard file formats, also known as universal file formats, may facilitate data handling within research groups and beyond, while ensuring that the data are readable and accessible over the time [34,35].

First, standard file formats ensure that most entities—such as a researcher’s future self, colleagues, or third parties—can process the files, whether the data consist of images, text files, data tables, or other types. Additionally, open file formats circumvent potential issues caused by closed proprietary file formats, which require certain software to open or process them. Not only do such propriety files potentially require paid software for processing, but long-term support for such software is also not guaranteed.

By using standard and open file formats, researchers ensure that their work remains accessible in the long term by avoiding dependencies. If certain tasks require software that use specific proprietary formats, it makes sense to keep this as the original file for long-term use, but to additionally consider producing a copy in a standard, open file format (Table 1). Best practices for formats vary by discipline and data type; for example, in bioinformatics, FASTA for DNA sequences ensures interoperability, while in health sciences, CSV for tabular data are supported by most statistical software.

**Table 1:** Exemplary file types and suggested formats for compatible and long-term preservation. [36].

File type	Suggested formats (digest) Format
Audio	• Free Lossless Audio Codec (FLAC) • Broadcast Wave (BWF) [version 0, 1 & 2] • MPEG-1/2 Audio Layer III (MP3)
Containers	• ZIP • Optical disc image (ISO) • Tape Archive (TAR)
Data tables (statistics)	• Comma Separated Value (CSV) • Plain text (ASCII Text version 7 bit, Unicode Text version UTF-8)
Text	• Portable Document Format/Archiving (PDF/A-1 and A-2) • Plain text (TXT), ASCII or UTF-8 encoding • OpenDocument Text (ODT) [version 1.0 to 1.3]
Images, Photographs	• Tagged Image File Format (TIFF) [versions 4 to 6] • JPEG 2000 Part 1 (JP2), lossless compression • Portable Network Graphics (PNG) [version 1.2] • JPEG (JPG) • Scalable Vector Graphics (SVG)
Video	• MPEG-4 (MP4) • Matroska Multimedia Video Container (MKV) • Material Exchange Format (MXF) • Codecs: FFV1; Advanced Video Coding (AVC), H.264, MPEG-4 Part 10; Uncompressed 4:2:2

We recognize that adopting open file formats may entail additional effort and careful handling to prevent information loss. Nevertheless, for ensuring data accessibility and long-term usability, open formats are essential.

## Rule 9: Share your research data

In the context of RDM, data publication refers to making research datasets openly accessible for review and reuse. In the context of the so-called reproducibility crisis [37], data sharing plays a key role to enable research reproducibility [38,39]. Research data publication has several advantages: it promotes transparency, credibility, long-term data accessibility, reproducibility, and collaboration. Data publication is a pillar of the Open Science movement and is considered the best practice in many research communities and subject domains. While in the past, researchers were hesitant to share their data, it is becoming more evident that researchers can also benefit from sharing their data through greater recognition of their scientific work.

**Fig 4:**
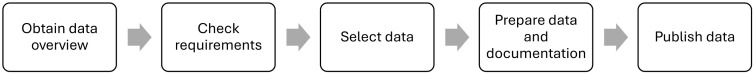
The process of data publication.

The FAIR guiding principles [40] are a set of guidelines for enhancing the reusability of scientific data. They stand for *Findable*, *Accessible*, *Interoperable*, and *Reusable*, emphasizing that data should be easily located, available for use, compatible across various systems, and useable for future research. The data publishing process can be divided into multiple steps (Fig 4).

In short, first obtain an overview of the data. Then, check requirements from funders, professional societies, and best practices (rules 1 and 3). Do not forget to consider any data policies or goals previously incorporated into the DMP (rule 7). Next, select the data to publish. Have clear criteria when selecting the data to publish (Table 2): although it is worthwhile publishing unique and publicly relevant data, avoid publishing test or discarded data, or data with no mid- and long-term relevance. However, always consider the protection of sensitive data, e.g., human subject data, and any steps required, such as de-identification techniques (reversible pseudonymization, irreversible anonymization) for personally identifiable information. For sensitive data, especially human subject data, sharing requires robust de-identification strategies to mitigate risks of re-identification while preserving data utility for research. This includes removing direct identifiers (e.g., names, addresses), aggregating variables (e.g., grouping ages into ranges, suppressing rare values, or adding noise to geographic data (jittering). These practices help balance openness with privacy protections under regulations like the Health Insurance Portability and Accountability Act (HIPAA) in the US or GDPR in the EU. Software tools (see S1 Table) may help with pseudonymization or anonymization, but alone do not guarantee thorough de-identification, and their outputs require strict monitoring. For legal certainty or orderly data de-identification, it is advisable to obtain expert oversight by contacting the legal department or the responsible data protection officer, respectively.

**Table 2:** Selected criteria in favor of and against the publication of research data.

Criteria in favor of data publication	Criteria opposed to data publication
** +** Data uniqueness	** **− Test data
** +** Data generation complexity	** **− Discarded and erroneous data
** +** Importance of data for the scientific community or the public.	** **− lack of medium- and long-term relevance
** **+ Data is free of personal data or is pseudonymized/anonymized	** **− Data contains personal information with the potential to identify an individual person

Once the data are selected for publication, prepare the data and metadata, including documentation, for publication. Check if the data requires further processing or needs conversion into an open format. Examine the metadata to see if it is adequate and complete. Then, choose an appropriate license (e.g., a Creative Commons [CC] or MIT-license) to specify how the data can be re-used by others [41]. As a rule of thumb, select a license that is as open as possible and imposes minimal restrictions.

Comparable to journal publications, data publications should be assigned a persistent identifier to enhance long-term findability and prevent isolation, in line with the FAIR principles. A unique Digital Object Identifier (DOI, https://www.doi.org/) is ideally provided by the data repository where the data are deposited, such as Zenodo (https://zenodo.org) or Dryad (https://datadryad.org). In addition to DOIs, identifiers for researchers (ORCID, https://orcid.org) and institutions (Research Organization Registry, ROR, https://ror.org/) facilitate clear communication of affiliations and allow others to reference properly.

Finally, publish the data. Choose a general-purpose data repository such as Zenodo and Dryad or select a topic-specific data repository. Use tools such as re3data (https://www.re3data.org) to search and identify a suitable data repository.

Researchers may face limited time or funding for data publication [42], but major funding agencies, such as the NSF and the DFG, provide support for data publication and associated fees. We note that publishing research data may also involve legal uncertainties, which could result in the decision not to share data. We recommend that researchers consult their institutional legal department to address any uncertainties or seek guidance from supraregional academic consulting services, where available (e.g., in Germany, services such as those provided by the Nationale Forschungsdateninfrastruktur (NFDI) e.V.).

## Rule 10: Continuous self-monitoring

Continuous self-monitoring is our final rule for effective RDM. This self-monitoring involves setting regular intervals at which to evaluate progress within and beyond projects in order to spot potential problems and ascertain the overall effectiveness of the work. By performing such self-monitoring, researchers can potentially mitigate the risk of getting lost in detail and better visualize goals.

Naturally, regular review allows mapping of progress against strategies, schedules, budgets, person-hours, and other metrics to keep work on track and allow for corrective actions, if required. This can help tackle issues that might be minor today but can compound over time and require excessive resources in the long run.

One aspect of self-assessment is not to be underestimated—it helps develop skills at the individual level and refine strategies at the organizational level. Researchers will be able to improve their understanding of what works best, evaluate management abilities, including RDM, and identify weak areas.

Hence, vigilantly tracking one’s research progress, assessing methodologies, and fostering personal growth are integral parts of efficient research management. It demands honesty, clarity, and the willingness to adapt and to make improvements whenever necessary.

## Discussion

In this manuscript, we provide ten simple rules for effective RDM, spanning external requirements, organization, methodology, documentation, and publication. These rules extend other project-specific RDM guidelines. Here, we also address RDM from the perspective of a university or other research institution, where research data accumulate from individual projects and must then be managed for reuse. In our view, the key aspect to effective RDM is investing in comprehensive documentation, as everything revolves around it: every action begins with a thought, and a documenting thought will eventually aid in organizing actions.

Although the offered ten rules address an effective RDM, they may vary by research field. Thus, we advise communicating and agreeing on such guidelines with project partners. Nonetheless, we believe they serve as a jump start for some and a valuable complement for others. We understand that individual researchers may have restricted means and limited experience to follow certain rules, such as habitually publishing data or to being mindful of all relevant legal issues of data publishing, including data protection or copyright, especially when compared to larger research groups with substantial funding. For the case of Germany, regional and supra-regional RDM consulting services are available for researchers, organized in state initiatives with focus on research data, such as the Landesinitiative Forschungsdaten (FDM-NDS) or the national society Nationale Forschungsdateninfrastruktur (NFDI) e.V.

In the realm of research data management (RDM), there are many stakeholders beyond funders and organizations promoting best practices. Potential conflicts may arise from stakeholders associated with the researcher’s facility, such as management or supervisors, or IT departments, concerning the extent of appreciation of RDM activities due to insufficient communication or agreements, such as a researcher asking for additional but unannounced computing power. Furthermore, research data professionals, data curators, or librarians typically mediate data preservation and access, can potentially result in conflicts over resource allocation or the choice of standards when interdisciplinary teams collaborate, e.g., in the case of metadata. Data protection officers, legal departments, and ethics committees introduce additional layers of administration, while privacy regulations (e.g., GDPR compliance) potentially contradict open data mandates (rule 9). This may result in data silos or delayed–or even suspended–sharing. To mitigate such issues, close cooperation and agreement among the stakeholders should be sought, including the establishment of mutual agreements, workflows, and policies (rule 1) and workflows.

We acknowledge the critical role of funders in enforcing RDM practices, such as data documentation and publication, to boost the potential impact of funding by enabling reuse possibilities through binding funding to said activities. This may be perceived as a top-down dependency of researchers, potentially in conflict with academic freedom, which is guarded by basic law in certain countries, such as Germany. However, the way a researcher documents and shares their methodology, data, and knowledge is a matter of research ethics and good scientific practice. We appreciate the growing efforts of funders, institutions, principal investigators, and researchers to both promote, support, and demand RDM practices. This extra effort will allow researchers to reap rewards such as well-organized work or recognition gained through citation of published data. We emphasize the overall benefit of orderly RDM to research, bolstering data quality, comprehensibility and reusability, and research credibility and reproducibility.

## Supporting information

S1 Table Selected software tools and platforms for Research Data Management. (PDF)

## References

[1] Batley J, Edwards D. Genome sequence data: management, storage, and visualization. Biotechniques. 2009;46(5):333–4, 336. doi: 10.2144/000113134 19480628

[2] Wetzstein G, Ozcan A, Gigan S, Fan S, Englund D, Soljačić M, et al. Inference in artificial intelligence with deep optics and photonics. Nature. 2020;588(7836):39–47. doi: 10.1038/s41586-020-2973-6 33268862

[3] Schwab S, Janiaud P, Dayan M, Amrhein V, Panczak R, Palagi PM, et al. Ten simple rules for good research practice. PLoS Comput Biol. 2022;18(6):e1010139. doi: 10.1371/journal.pcbi.1010139 35737655 PMC9223329

[4] Wilson G, Bryan J, Cranston K, Kitzes J, Nederbragt L, Teal TK. Good enough practices in scientific computing. PLOS Comput Biol. 2017;13(6):e1005510. doi: 10.1371/journal.pcbi.1005510 PMC5480810 28640806

[5] Deutsche Forschungsgemeinschaft. Guidelines for Safeguarding Good Research Practice. Code of Conduct. 2022 Apr 20 [cited 2024 May 31]; Available from: https://zenodo.org/record/3923601

[6] Gomez-Diaz T, Recio T. Research software vs. research data I: towards a research data definition in the open science context. F1000Res. 2022;11:118. doi: 10.12688/f1000research.78195.2 36415208 PMC9650106

[7] Surkis A, Read K. Research data management. J Med Libr Assoc. 2015;103(3):154–6. doi: 10.3163/1536-5050.103.3.011 26213510 PMC4511058

[8] Tayefi M, Ngo P, Chomutare T, Dalianis H, Salvi E, Budrionis A, et al. Challenges and opportunities beyond structured data in analysis of electronic health records. WIREs Comput Stats. 2021;13(6). doi: 10.1002/wics.1549

[9] Ikotun AM, Ezugwu AE, Abualigah L, Abuhaija B, Heming J. K-means clustering algorithms: a comprehensive review, variants analysis, and advances in the era of big data. Inf Sci. 2023;622:178–210. 10.1016/j.ins.2022.11.139

[10] Krepel J, Kircher M, Kohls M, Jung K. Comparison of merging strategies for building machine learning models on multiple independent gene expression data sets. Stat Anal Data Min ASA Data Sci J. 2022;15(1):112–24. doi: 10.1002/sam.11549

[11] Winter C, Kosch R, Ludlow M, Osterhaus ADME, Jung K. Network meta-analysis correlates with analysis of merged independent transcriptome expression data. BMC Bioinform. 2019;20(1):144. doi: 10.1186/s12859-019-2705-9 30876387 PMC6420731

[12] Debray TPA, Moons KGM, van Valkenhoef G, Efthimiou O, Hummel N, Groenwold RHH, et al. Get real in individual participant data (IPD) meta-analysis: a review of the methodology. Res Synth Methods. 2015;6(4):293–309. doi: 10.1002/jrsm.1160 26287812 PMC5042043

[13] Randles WGL. The evaluation of columbus’ ‘India’ project by Portuguese and Spanish cosmographers in the light of the geographical science of the period. Imago Mundi. 1990;42(1):50–64. doi: 10.1080/03085699008592691

[14] Michener WK. Ten simple rules for creating a good data management plan. PLoS Comput Biol. 2015;11(10):e1004525. doi: 10.1371/journal.pcbi.1004525 26492633 PMC4619636

[15] Sandve GK, Nekrutenko A, Taylor J, Hovig E. Ten simple rules for reproducible computational research. PLoS Comput Biol. 2013;9(10):e1003285. doi: 10.1371/journal.pcbi.1003285 24204232 PMC3812051

[16] Fungtammasan A, Lee A, Taroni J, Wheeler K, Chin C-S, Davis S, et al. Ten simple rules for large-scale data processing. PLoS Comput Biol. 2022;18(2):e1009757. doi: 10.1371/journal.pcbi.1009757 35143491 PMC8830682

[17] Fischer BA, Zigmond MJ. The essential nature of sharing in science. Sci Eng Ethics. 2010;16(4):783–99. 10.1007/s11948-010-9239-x 21108019

[18] Duke CS, Porter JH. The ethics of data sharing and reuse in biology. BioScience. 2013;63(6):483–9. doi: 10.1525/bio.2013.63.6.10

[19] Ramachandran R, Bugbee K, Murphy K. From open data to open science. Earth Space Sci. 2021;8(5):e2020EA001562. 10.1029/2020EA001562

[20] National Research Council. Sharing publication-related data and materials: responsibilities of authorship in the life sciences. Washington, D.C.: National Academies Press; 2003. Available from: 10.17226/10613 22649805

[21] Savage CJ, Vickers AJ. Empirical study of data sharing by authors publishing in PLoS journals. PLoS One. 2009;4(9):e7078. doi: 10.1371/journal.pone.0007078 19763261 PMC2739314

[22] Kanza S, Knight NJ. Behind every great research project is great data management. BMC Res Notes. 2022;15(1):20. doi: 10.1186/s13104-022-05908-5 35063017 PMC8781028

[23] Council for International Organizations of Medical Sciences CIOMS. International ethical guidelines for health-related research involving humans. 4th ed. Geneva: Council for International Organizations of Medical Sciences; 2016. Available from: https://www.who.int/docs/default-source/ethics/web-cioms-ethicalguidelines.pdf?sfvrsn=f62ee074_0 40523065

[24] World Conferences on Research Integrity. Singapore Statement [Internet]. [cited 2025 Jan 6]. Available from: https://www.wcrif.org/statement

[25] Koltay T. Data governance, data literacy and the management of data quality. IFLA J. 2016;42(4):303–12. doi: 10.1177/0340035216672238

[26] Sargiotis D. Data governance policies and standards: development and implementation. In: Data governance. Cham: Springer; 2024. p. 247–77. Available from: 10.1007/978-3-031-67268-2_7

[27] Ladley J. Data governance: how to design, deploy and sustain an effective data governance program. 2nd ed. Academic Press; 2019. 10.1016/C2017-0-03353-0

[28] Carroll MW. Sharing research data and intellectual property law: a primer. PLoS Biol. 2015;13(8):e1002235. doi: 10.1371/journal.pbio.1002235 26313685 PMC4551669

[29] UK Medical Research Council (UKRI). Regulatory Support Centre. Retention framework for research data and records [Internet]. 2022 [cited 2025 Jan 27]. Available from: https://www.ukri.org/publications/retention-framework-for-research-data-and-records/

[30] Ryan TP. Sample size determination and power. Hoboken, NJ: John Wiley & Sons; 2013. 10.1002/9781118439241

[31] Hassenstein MJ, Vanella P. Data quality—concepts and problems. Encyclopedia. 2022;2(1):498–510. 10.3390/encyclopedia2010032

[32] Buchanan EM, Crain SE, Cunningham AL, Johnson HR, Stash H, Papadatou-Pastou M, et al. Getting started creating data dictionaries: how to create a shareable data set. Adv Methods Pract Psychol Sci. 2021;4(1):2515245920928007. doi: 10.1177/2515245920928007

[33] Deutsche Forschungsgemeinschaft (DFG). Umgang mit Forschungsdaten. Checkliste für Antragstellende zur Planung und zur Beschreibung des Umgangs mit Forschungsdaten in Forschungsvorhaben. [Internet]; 2021. Available from: https://www.dfg.de/resource/blob/174732/3c6343eed2054edc0d184edff9786044/forschungsdaten-checkliste-de-data.pdf

[34] McKiernan EC, Barba L, Bourne PE, Carter C, Chandler Z, Choudhury S, et al. Policy recommendations to ensure that research software is openly accessible and reusable. PLoS Biol. 2023;21(7):e3002204. doi: 10.1371/journal.pbio.3002204 37478129 PMC10396347

[35] Wilson M, Tchantchaleishvili V. The importance of free and open source software and open standards in modern scientific publishing. Publications. 2013;1(2):49–55. doi: 10.3390/publications1020049

[36] Open Preservation Foundation. International comparison of recommended file formats (v1.3) [Internet]. 2023. Available from: https://openpreservation.org/resources/member-groups/international-comparison-of-recommended-file-formats/

[37] Baker M. 1,500 scientists lift the lid on reproducibility. Nature. 2016;533(7604):452–4. doi: 10.1038/533452a 27225100

[38] Miyakawa T. No raw data, no science: another possible source of the reproducibility crisis. Mol Brain. 2020;13(1):24. doi: 10.1186/s13041-020-0552-2 32079532 PMC7033918

[39] Peng RD. Reproducible research in computational science. Science. 2011;334(6060):1226–7. 10.1126/science.1213847 22144613 PMC3383002

[40] Wilkinson MD, Dumontier M, Aalbersberg IJJ, Appleton G, Axton M, Baak A, et al. The FAIR Guiding Principles for scientific data management and stewardship. Sci Data. 2016;3:160018. doi: 10.1038/sdata.2016.18 26978244 PMC4792175

[41] Labastida I, Margoni T. Licensing FAIR data for reuse. Data Intell. 2020;2(1–2):199–207. doi: 10.1162/dint_a_00042

[42] Tenopir C, Allard S, Douglass K, Aydinoglu AU, Wu L, Read E, et al. Data sharing by scientists: practices and perceptions. PLoS One. 2011;6(6):e21101. doi: 10.1371/journal.pone.0021101 21738610 PMC3126798

