# Supplementary material for: Ten simple rules for effective research data management
Source: PLoS Comput Biol. 2025 Dec 8;21(12):e1013779. doi: 10.1371/journal.pcbi.1013779 (PMC12685206; doi:10.1371/journal.pcbi.1013779)
Supplement: S1 Table — (PDF) [file pcbi.1013779.s001.pdf]

Supplementary material: Ten Simple Rules for Effective Research Data Management

Max J. Hassenstein[1,2 *] and Klaus Jung[1]

[1] Institute of Animal Genomics, University of Veterinary Medicine Hannover, Foundation, Lower Saxony, Germany

[2] Unit for Research Data Management, University of Veterinary Medicine Hannover, Foundation, Lower Saxony, Germany

* Corresponding author; E-mail: max.hassenstein@tiho-hannover.de (MJH)

Table S1. Selected software tools and platforms for Research Data Management

Aim, Task	Tool or platform	Provision	References
Data anonymization	Amnesia	online	https://amnesia.openaire.eu
	ARX Data Anonymization Tool	local	https://arx.deidentifier.org; https://github.com/arx-deidentifier/arx
	Presidio	local	https://github.com/microsoft/presidio
	sdcMicro: Statistical Disclosure Control Methods for Anonymization of Data and Risk Estimation (R Package)	local	https://doi.org/10.18637/jss.v067.i04; https://cran.r-project.org/web/packages/sdcMicro/index.html
Data backup	Borg	local	https://www.borgbackup.org; https://github.com/borgbackup
	Duplicati	local	https://duplicati.com
	Rclone	local	https://rclone.org
Data management and collection	FAIRDOM-SEEK	local	https://seek4science.org; https://github.com/seek4science/seek
	RedCap	local	https://project-redcap.org
Data management plan	ARGOS	online	https://argos.openaire.eu
	DMPonline	online	https://dmponline.dcc.ac.uk; https://github.com/DMPRoadmap
	RDMO (Research data management organiser)	online, local	https://rdmorganiser.github.io; https://github.com/rdmorganiser
Data exchange	AcademicCloud (online service hosted at GWDG Göttingen) [Available for German research organizations]	online	https://academiccloud.de
	GigaMove (hosted at RWTH Aachen) [Available for German research organizations]	online	https://gigamove.rwth-aachen.de
	Globus	online	https://www.globus.org
	Nextcloud	local	https://nextcloud.com
Data wrangling, transformation	OpenRefine	local	https://openrefine.org; https://github.com/OpenRefine
	Python, Jupyter Notebook (IDE)	local	https://www.python.org; https://jupyter.org
	R, RStudio (IDE)	local	https://www.r-project.org; https://posit.co

Table S1. Selected software tools and platforms for Research Data Management (*continued*)

Aim, Task	Tool or platform	Provision	References
Electronic Laboratory Notebook (ELN)	eLabFTW	local	https://www.elabftw.net
	Chemotion	local	https://chemotion.net
Encryption	VeraCrypt	local	https://www.veracrypt.fr
File naming	Automator (macOS)	local	https://support.apple.com/guide/automator/welcome/mac
	Ant renamer (Linux)	local	https://www.antp.be/software/renamer
	PowerRename (Windows)	local	https://learn.microsoft.com/en-us/windows/powertoys/powerrename
Project management	OpenProject	local	https://www.openproject.org
Publication	Dryad (Repository)	online	https://datadryad.org
	Figshare (Repository)	online	https://figshare.com
	RADAR (Repository)	online	https://www.radar-service.eu
	Re3data (Registry of research data repositories)	online	https://re3data.org
	Zenodo (Repository)	online	https://zenodo.org
Statistics and Data Science	JASP	local	https://jasp-stats.org
	Python, Jupyter Notebook (IDE)	local	https://www.python.org; https://jupyter.org
	R, RStudio (IDE)	local	https://www.r-project.org; https://posit.co
Version control, development	Git	local	https://git-scm.com
	GitHub	online	https://github.com
	GitLab (also hosted at GWDG Göttingen) [Available for German research organizations]	local, online	https://gitlab.com; https://gitlab.gwdg.de
